# Is There a Need for Functional Radiographs in Diagnosing Lumbar Instability?

**DOI:** 10.1177/21925682241306025

**Published:** 2024-12-09

**Authors:** Marie-Christine Lutschounig, Irene Katharina Sigmund, Irene Steiner, Anna Rienmüller, Christoph Stihsen, Reinhard Windhager, Josef Georg Grohs

**Affiliations:** 1 Department of Orthopedics, 27271 Medical University of Vienna, Vienna, Austria; 2 CeDAS, Institute of Medical Statistics, 27271 Medical University of Vienna, Vienna, Austria

**Keywords:** functional radiography, segmental instability, sagittal translation, sagittal rotation, spondylolisthesis, lumbar spine

## Abstract

**Study Design:**

Retrospective radiological database analysis.

**Objective:**

The aim of this study was to assess the value of functional radiography (FRF = flexion; FRE = extension) compared to MRI and standing sagittal plane full spine radiography (SP) with low-grade spondylolisthesis.

**Methods:**

Sagittal translation (ST) and rotation (SR) were measured between all lumbar levels to assess instability. The differences for ST and SR of SP and FRE as well as MRI and FRF were calculated. In addition, the lumbar lordosis, the sacral slope, the pelvic tilt and the pelvic incidence were measured.

**Results:**

Radiological datasets of 55 patients with 165 lumbar segments fulfilled inclusion criteria. Instability was diagnosed in 20 segments (12.1%) with SP/MRI compared to 14 segments (8.5%) using FRF/FRE with ST. SR functional radiographs showed instability in 41 segments (25%) and 23 segments (14%) using SP/MRI. The intraclass correlation coefficients (ICC) of ST between SP and FRE for L3/L4, L4/L5, and L5/S1 were 0.74, 0.84 and 0.97, respectively, indicating moderate to excellent agreement between imaging methods. For SP/FRE, the ICCs of the SR were 0.72, 0.61 and 0.64, respectively with moderate agreement. The ICCs of the ST for L3/4, L4/5, and L5/S1 showed moderate to good agreement between MRI and FRF with values of 0.52, 0.77, and 0.80, respectively. Regarding SR, poor agreement between MRI and FRF was observed. The ICCs for L3/4, L4/5, L5/S1 were 0.16, 0.23 and 0.23.

**Conclusion:**

Based on our results, instability may also be diagnosed by calculating the difference in the ST in SP and MRI without additional functional radiographs. However, FRF showed translational instability more clearly than MRI in some patients and might still be an asset in borderline cases.

## Introduction

Patients with degenerative and true spondylolisthesis of the lumbar spine and low back pain with or without radiculopathy may require spinal surgery. However, diagnosing vertebral instability remains challenging for spine surgeons and continues to generate considerable debate. While lumbar spine instability is defined as abnormal lumbar motion during physiological loading of the spine,^1-3^ there is no universally accepted diagnostic standard for identifying vertebral instability in patients with low back pain and/or radiculopathy.^4^ Various imaging modalities are available, including full spine radiography in the sagittal plane in a standing position (SP), flexion (FRF) and extension (FRE) radiographs, computed tomography (CT) scans and magnetic resonance imaging (MRI) in a recumbent supine, psoas-relaxed position. Due to simple usage, availability, and cost-effectiveness, flexion and extension radiography are commonly applied in clinical routine to detect pathologic lumbar mobility.^1,5-7^ These diagnostic tests are recommended in a standing inclined and reclined position of the patient’s spine.^8-10^ Although functional X-rays remain the most common diagnostic technique to evaluate sagittal instability,^11,12^ they have never been widely accepted.^13^ In addition, MRI was not readily available when functional radiographs were introduced.

However, due to the lack of uniform reference standards, no guideline exists for the most appropriate imaging method to detect lumbar vertebral instability.

The necessity of functional radiography for diagnosing lumbar instability is still discussed in the literature.^13,14^ There are few studies comparing the efficacy of functional radiographs vs standing sagittal plane radiographs and MRI in the diagnosis of lumbar instability. As there is no consensus, the ideal imaging method is not yet known.

This study aims to assess the value of flexion-extension radiography for diagnosing segmental instability in patients with spondylolisthesis by comparing full spine radiography with functional radiography in extension and conventional MRI to functional radiography in flexion. Additionally, the reliability of the radiographic anthropometric parameters of sagittal translation and sagittal rotation is examined.

## Material & Methods

### Study design

This retrospective study was conducted at a tertiary healthcare center providing advanced specialist treatment of spinal disorders. Radiological images of patients suffering from low back pain due to true spondylolisthesis or degenerative spondylolisthesis (pseudolisthesis) were analyzed. The study was approved by the institutional review board of the Medical University of Vienna, Austria (EK 1835/2016) and conducted in accordance with the Declaration of Helsinki.^15^

As this is a retrospective radiological database analysis and the patients experience neither direct benefit nor harm from the study, no informed consent was obtained.

### Study Population

**Figure 1.**
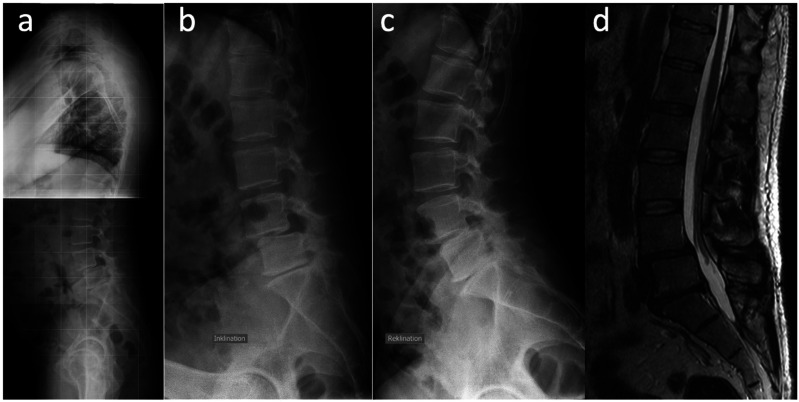
Images of a 48-year-old male patient with a degenerative spondylolisthesis in L4/5 Meyerding II. (a) radiography in sagittal plane of the full spine (SP) in a standing position, (b) flexion (FRF), (c) extension radiography (FRE) in a standing position, and (d) T2-weighted MRI in a recumbent supine, psoas-relaxed position.

Patients older than 18 years with low back pain and low-grade spondylolisthesis (Grades 1 and 2 according to Meyerding’s classification^16^) planned for lumbar interbody fusion were eligible for inclusion. Standardized diagnostic imaging methods had to be available: Standing full spine radiography in the sagittal plane (SP), a flexion (FRF = functional radiography flexion) and extension radiography (FRE = functional radiography extension) of the lumbar spine in a standing position, and an MRI of the lumbar spine in a recumbent supine position (Figure 1). Patients with X-ray imaging carried out externally of the tertiary health care center, incomplete dataset (missing functional radiography, full spine radiography or MRI) or twisted imaging material were excluded.

### Assessment of Anthropometric Parameters

**Figure 2.**
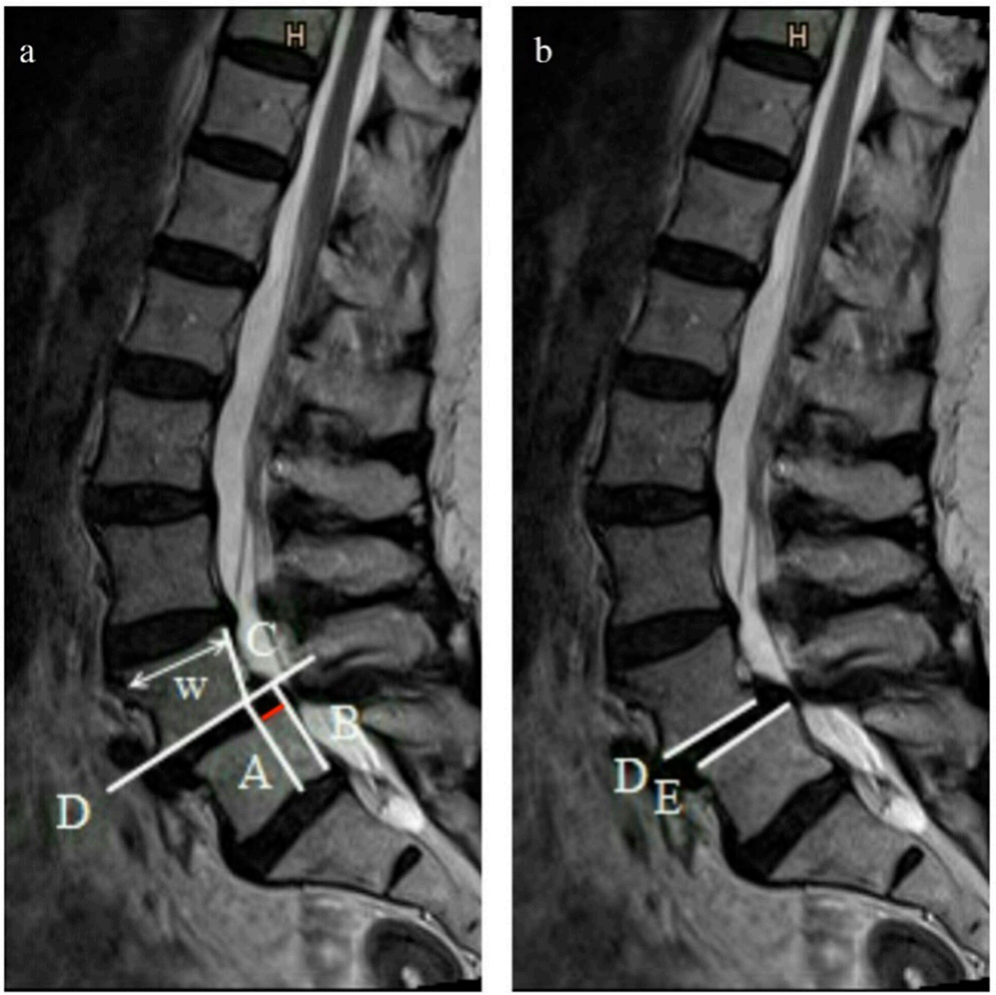
Measurement technique in a sagittal T2-weighted MRI: in figure a, the red line is the distance between line A and B and represents the sagittal translation (ST). The sagittal rotation (SR) is the angle between line D and E shown in figure b.

To ensure a constant magnification factor of the X-rays, SP, FRE, and FRF were performed in our hospital. The radiographs and the MRI were digitized, and all measurements were done in the radiology information system IMPAX EE R20 XIX (Agfa HealthCare GmbH, Mortsel, Belgium). For each of the four images, the sagittal translation (ST) and sagittal rotation (SR) were measured according to Dupuis et al^1^ Figure 2 shows the measurement technique performed in the present study. The distance between the line drawn along the trailing edge of the inferior vertebral body (line B, Figure 2A), the parallel line (line A, Figure 2A) in the extension of the intersection of the line along the endplate of the superior vertebral body (line D, Figure 2A) and the line along the trailing edge of the superior vertebral body (line C, Figure 2A) were determined. This distance represents the sagittal translation and was measured in absolute values and percentage of the subsequent vertebral body’s upper plate width (w, Figure 2A). The angle between the line along the low endplate of the superior vertebral body (line D, Figure 2B) and the upper plate of the inferior vertebral body (line E, Figure 2B) measured in degrees represented the sagittal rotation.

Generally, instability is determined by calculating the absolute difference of the ST between FRF and FRE and was, hence, calculated in this study.^17^ During an MRI in a lying position, the hips and the lumbar spine of elderly patients may also be unduly flexed. Additionally, the spine of elderly patients may be excessively extended during radiography in the sagittal plane of the full spine in a standing position. Therefore, the difference between the ST of FRE and SP and between the ST of FRF and MRI were calculated as well as the difference between the SR of FRE and SP and between the SR of FRF and MRI. Since degenerative spondylolisthesis is a progressive disease, the time interval between functional X-ray and MRI was also calculated.

A lumbar instability is universally defined as sagittal translation ≥4 mm or a sagittal rotation of ≥8°.^1,18-21^ Using both definitions, the results were compared between functional X-rays and SP/MRI. To avoid inaccuracies caused by magnifications, the percentage of the upper body width was also calculated (relative value).

In addition, the sagittal plane was calculated in SP: The lumbar lordosis, the sacral slope, the pelvic tilt and the pelvic incidence were measured as described in the study by Vialle et al.^22^

### Statistical Analysis

Descriptive analysis includes minimum, maximum, median, first and third quartile (Q1, Q3), mean, standard deviation of quantitative variables, and absolute and relative frequencies of categorical variables.

Agreement between SP and FRE, and between MRI and FRF, was assessed graphically using Bland Altman plots displaying the mean of the two methods on the horizontal axis and the differences of the methods on the vertical axis. The so-called 95% limits of agreement (defined as mean difference +/−1.96*standard deviation of the difference) were calculated and illustrated in the plot. The mean difference with a 95% confidence interval (95% CI) was calculated, and a one-sample t-test (H_0_: the mean difference between the two methods equals zero) was conducted. The ICC with 95% confidence limits is reported. The ICC was calculated by variance component analysis (SAS Proc varcomp, R-package irr: R-function ICC), whereby patient-ID and method were taken as random factors, and the REML estimation was used. The ICC was then calculated as the proportion of the estimated variance component of the patient divided by the total variability. Comparison of instability (dichotomous) between functional radiographs and SP/MRI was done by cross-tabulation and McNemar-tests. The two-sided significance level has been set to alpha = 0.05. Statistical analyses were carried out with SAS 9.4. and R 4.2.2.

## Results

### Patients and Characteristics

Between January 2013 and August 2017, 76 patients with low back pain, spondylolisthesis and a full imaging dataset were recorded. Five patients were excluded because a spondylolisthesis Grade 3 or 4 was present, according to Meyerding’s classification. In 16 patients, measurements were impossible due to unwanted lumbar spine rotation in functional radiographs, the presence of transitional vertebrae or significant scoliosis. Overall, 55 patients with an evaluable dataset and planned spinal fusion (Transforaminal Lumbar Interbody Fusion) due to low back pain and a true spondylolisthesis (n = 7, 12.7%) or degenerative spondylolisthesis (n = 48, 87.3%) were eligible for inclusion. Four women (n = 4/7; 57.1%) and three men (n = 3/7; 42.9%) were diagnosed with spondylolisthesis vera with a median age of 49 years (range: 34-59 years). A degenerative pseudolisthesis was diagnosed in 31 women (n = 31/48; 64.6%) and 17 men (n = 17/48; 35.4%). The median age in the degenerative group was 63 years (range: 39-82 years). Table 1 shows the descriptive statistics of the ST and the SR between the measured vertebral segments. The level of interest was L3/L4 in nine patients (16.4%), in 29 patients (52.7%) L4/L5 and in another 16 patients (29.1%) L5/S1. In one patient, multiple segments (L4 to S1) were of interest (1.8%). 46 patients (83.6%) had a Grade 1, and nine patients (16.4%) had a Grade 2 spondylolisthesis according to Meyerding’s classification.^16^ The mean C7 plumb line was 36 mm (±49; −44-212 mm). The mean lumbar lordosis, sacral slope, pelvic tilt, and pelvic incidence were 52.2° (±14°; 13-78°), 33.9° (±9°; 11-56°), 21° (±9°; 0-48°), and 54.9° (±13°; 28-85°), respectively.

**Table 1.** Descriptive statistics. The ST = Sagittal translation (mm) and SR = Sagittal rotation (°) between the measured vertebral bodies are calculated. FRF = Functional radiography flexion, FRE = Functional radiography extension, MRI = Magnetic Resonance Imaging, SP = Radiography in sagittal plane of the full spine in a standing position.

Radiography	Variable	Minimum	Q1	Median	Q3	Maximum	Mean	SD (±)
FRF	ST (mm)							
	L3/L4	−3.0	0.0	0.0	2.0	11.0	1.0	2.6
	L4/L5	−3.0	0.0	1.0	8.0	12.0	3.4	4.3
	L5/S1	−6.0	0.0	0.0	0.0	23.0	2.0	5.5
	SR (°)							
	L3/L4	−8.0	0.2	1.1	2.1	12.0	1.4	2.9
	L4/L5	−6.0	−0.2	0.8	3.2	10.6	1.2	3.4
	L5/S1	−5.0	0.9	3.0	6.9	11.5	3.4	3.8
FRE	ST (mm)							
	L3/L4	−5.0	0.0	0.0	0.0	7.0	0.1	2.3
	L4/L5	−2.0	0.0	1.0	5.5	14.0	2.8	3.9
	L5/S1	−6.0	0.0	0.0	0.0	23.0	1.7	5.1
	SR (°)							
	L3/L4	0.5	5.8	7.9	9.0	18.0	7.6	3.6
	L4/L5	−1.7	1.9	4.8	9.7	21.0	6.0	5.3
	L5/S1	0.0	4.0	7.2	11.0	28.9	8.2	6.1
MRI	ST (mm)							
	L3/L4	−4.0	0.0	0.0	0.0	5.0	0.2	1.6
	L4/L5	−3.0	0.0	1.0	5.0	9.0	2.3	3.5
	L5/S1	−7.0	−2.0	0.0	0.0	13.0	0.4	3.9
	SR (°)							
	L3/L4	−0.9	2.7	4.9	7.2	11	4.9	3.1
	L4/L5	−9.0	2.8	4.4	7.9	13.8	4.9	4.6
	L5/S1	0.0	4.4	9.6	12.4	16.4	8.6	5.1
SP	ST (mm)							
	L3/L4	−5.0	0.0	0.0	0.0	8.0	0.3	2.1
	L4/L5	−3.0	0.0	0.0	6.0	14.0	3.1	4.2
	L5/S1	−6.0	0.0	0.0	0.0	23.0	1.8	5.3
	SR (°)							
	L3/L4	0.0	3.7	8.0	10.9	15.1	7.4	4.5
	L4/L5	−4.9	1.0	5.0	7.9	16.0	4.9	4.9
	L5/S1	−10.0	3.9	6.9	9.9	22.1	7.2	5.6

The average time interval between functional radiographs and MRI was 3.8 months (± 4.7; −16-19).

### Sagittal Translation

**Figure 3.**
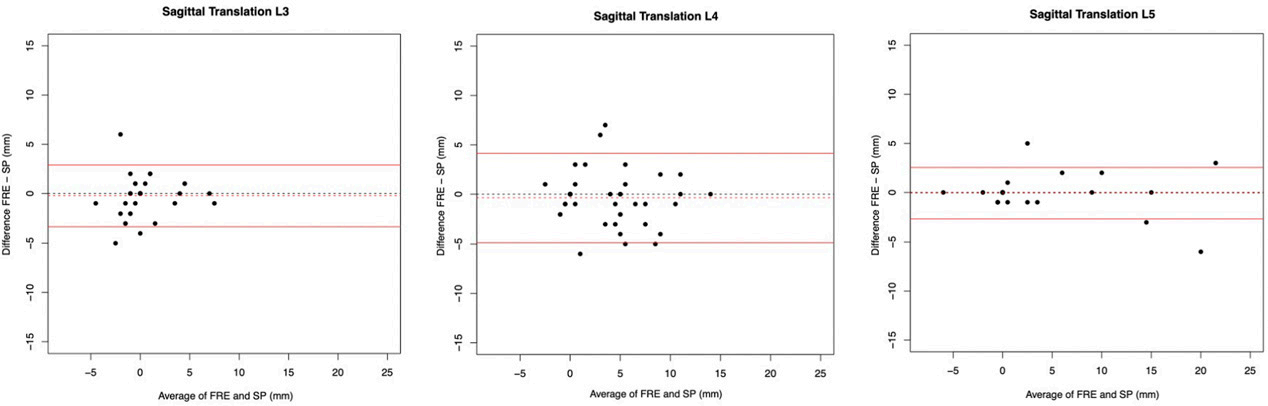
Bland Altman plots to represent the agreement of measurements of L3/4, L4/L5 and L5/S1 for sagittal translation between the FRE (functional radiography extension) and SP (radiography in sagittal plane of the full spine).

Intraclass correlation coefficients, the mean difference, the 95% limits of agreement of the Bland Altman plots and their relative values of the FRE and SP measurements regarding sagittal translation are given in Table 2. Moderate agreement for sagittal translation for the vertebral level L3/L4, good agreement for L4/5, and excellent agreement for L5/S1 between both imaging methods were calculated with ICCs. The ICC values for L3/L4, L4/L5 and L5/S1 were 0.74 [0.59; 0.84], 0.84 [0.74; 0.90] and 0.97 [0.95; 0.98], respectively. In the Bland Altman plots (Figure 3) of the sagittal translation L3/L4, L4/L5 and L5/S1, no statistically significant difference between the two images (FRE and SP) was seen (one-sample t-tests: *P* = 0.31/ *P* = 0.25/ *P* = 0.76).

**Table 2.** Intraclass Correlations (ICC), mean differences, and 95% limits of agreement (LoA) for sagittal translation, their relative values, and sagittal rotation of the FRE (functional radiography extension) vs SP (radiography in sagittal plane of the full spine). In parenthesis, the 95% confidence interval is presented.

FRE vs SP	ICC	Mean difference	LoA
Sagittal Translation			
L3/4	0.74 (0.6; 0.84)	−0.22 (−0.65; 0.21)	−3.32 – 2.89
L4/5	0.84 (0.74; 0.9)	−0.36 (−0.98; 0.26)	−4.86 – 4.14
L5/S1	0.97 (0.95; 0.98)	−0.06 (−0.41; 0.3)	−2.65 – 2.54
Relative Values			
L3/L4	0.75 (0.6; 0.84)	−0.53 (−1.48, 0.43)	−7.46 – 6.4
L4/L5	0.84 (0.74; 0.9)	−1.08 (−2.62; 0.47)	−12.28 – 10.12
L5/S1	0.97 (0.94; 0.98)	−0.17 (−1.06; 0.72)	−6.61 – 6.27
Sagittal Rotation			
L3/4	0.72 (0.57; 0.83)	0.24 (−0.58; 1.05)	−5.68 – 6.14
L4/5	0.61 (0.42; 0.75)	1.04 (−0.16; 2.23)	−7.61 – 9.69
L5/S1	0.64 (0.46; 0.78)	1.07 (−0.25; 2.39)	−8.49 – 10.63

However, measurements of FRE were, on average, lower than in the SP. The mean differences ranged between 0.06 mm and 0.36 mm. The limits of agreement (LoA) of L4/L5 (−4.86 to 4.14 mm) were noticeably larger than those of L3/4 (−3.32 to 2.89 mm) and L5/S1 (−2.65 to 2.54 mm).

**Figure 4.**
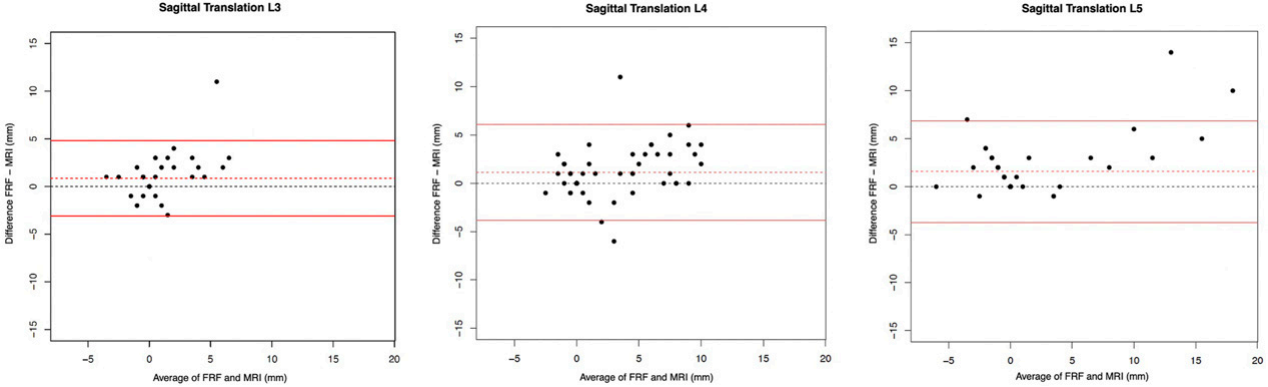
Bland Altman plots to represent the agreement of measurements of L3/4, L4/L5 and L5/S1 for sagittal translation between the FRF (functional radiography flexion) and MRI (Magnetic Resonance Imaging).

Table 3 summarizes the ICCs, the mean difference and the 95% limits of agreement of the Bland Altman plots (Figure 4) of the sagittal translations measured in the FRF and MRI, and corresponding relative values. The ICCs of the sagittal translation of L3/L4 (0.52 [0.28; 0.70]) showed moderate agreement between FRF and MRI measurements, while the ICCs of L4/5 (0.76 [0.59; 0.87]) and L5/S1 (0.8 [0.57; 0.90]) revealed good correlation.^23^ Regarding the sagittal translation of L3/4, L4/5 and L5/S1, the FRF gave statistically significantly higher values than the MRI (*P* = 0.003/ *P* = 0.002/ *P* < 0.0001).

**Table 3.** Intraclass Correlations (ICC), mean differences, and 95% limits of agreement (LoA) for sagittal translation, their relative values, and the sagittal rotation of the FRF (functional radiography flexion) vs MRI (Magnetic Resonance Imaging). In parenthesis, the 95% confidence interval is presented.

FRF vs MRI	ICC	Mean difference	LoA
Sagittal Translation			
L3/4	0.52 (0.28; 0.7)	0.86 (0.31; 1.40)	−3.11 – 4.82
L4/5	0.76 (0.59; 0.87)	1.13 (0.45; 1.81)	−3.82 – 6.08
L5/S1	0.8 (0.57; 0.9)	1.58 (0.85; 2.31)	−3.72 – 6.89
Relative values			
L3/L4	0.56 (0.33; 0.72)	1.87 (0.58; 3.16)	−7.48 – 11.22
L4/5	0.83 (0.72; 0.9)	1.51 (−0.12; 3.14)	−10.29 – 13.31
L5/S1	0.85 (0.67; 0.93)	3.67 (1.94; 5.39)	−8.82 – 16.15
Sagittal Rotation			
L3/4	0.16 (−0.07; 0.39)	−3.49 (−4.49; −2.52)	−10.69 – 3.71
L4/5	0.23 (−0.03; 0.47)	−3.64 (−4.92; −2.37)	−12.88 – 5.59
L5/S1	0.23 (−0.07; 0.5)	−5.2 (−6.55; −3.85)	−14.99 – 4.58

For FRF and MRI measurements, the LoA were rather large (from −3.11 to 6.89 mm). The mean differences were between 0.86 and 1.58.

### Sagittal Rotation

**Figure 5.**
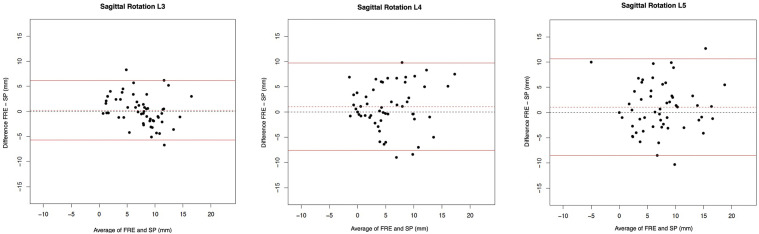
Bland Altman plots to represent the agreement of measurements of L3/4, L4/5 and L5/S1 for sagittal rotation between the FRE (functional radiography extension) and SP (radiography in sagittal plane of the full spine).

Table 2 shows the ICCs, mean difference and 95% limits of agreement of the Bland Altman plots (Figure 5) of the sagittal rotation measured on FRE and SP measurements, and corresponding relative values. Moderate agreement of the sagittal rotation between the FRE and SP with ICC values of L3/L4 (0.72 [0.57; 0.83]), L4/5 (0.61 [0.42; 0.75]) and L5/S1 (0.64 [0.46; 0.78]) was seen. Measurements of the FRE were, on average, higher than of SP, but no statistically significant difference between both imaging modalities was observed (mean differences ranged from 0.24° to 1.1°). LoA values ranged from −5.68 to 10.63 (L3/4: −5.68-6.14; L4/5: −7.61-9.69; L5/S1: −8.49-10.63). No substantial bias could be found. However, the variability of the differences was quite large, which was reflected by the broad range of the limits of agreement.

**Figure 6.**
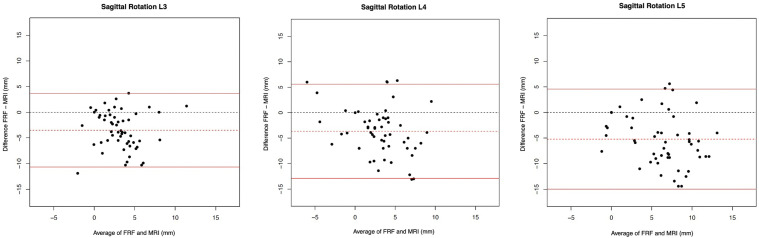
Bland Altman plots to represent the agreement of measurements of L3/4, L4/5 and L5/S1 for sagittal rotation between the FRF (functional radiography flexion) and MRI (Magnetic Resonance Imaging).

ICCs, the mean difference and the 95% limits of agreement of the Bland Altman plots (Figure 6) of the FRF and MRI measurements for sagittal rotation are given in Table 3. Poor agreement of the sagittal rotation between the FRF and MRI was shown for all lumbar levels with ICC values of L3/L4 (0.16 [−0.07; 0.39]), L4/L5 (0.23 [−0.03; 0.47]) and L5/S1 (0.23 [−0.07; 0.5]). For all three levels, mean differences were below zero with lower values of FRF compared to MRI (*P* < 0.0001 for L3/4, L4/5 and L5/S1). Therefore, some bias seemed to be present. The range of the limits of agreement for L3/4, L4/5 and L5/S1 were −10.69-3.71, −12.88-5.59 and −14.99-4.58, respectively.

### Instability

When using the commonly accepted classification for lumbar instability (ST ≥4 mm or SR ≥8° between FRE and FRF), instability was detected in 14 (8.5%) out of 165 levels in 55 patients according to the differences in the sagittal translation between FRE and FRF, and in 41 (25%) lumbar segments according to the sagittal rotation. If the same classification of instability was applied between SP and MRI, instability was diagnosed in 20 (12.1%) and 24 (15%) segments according to the differences in the sagittal translation and rotation, respectively (Table 4). Out of the 29 segments where instability according to ST was detected with either FRE/FRF or SP/MRI, five segments were detected with both methods, 15 segments only with SP/MRI and nine segments only with FRE/FRF. For SR, out of the 58 segments where instability was detected with either FRE/FRF or SP/MRI, seven segments were detected with both methods, 17 segments only with SP/MRI and 34 segments only with FRE/FRF. There was no statistically significant difference seen when comparing ST between functional radiographs and SP/MRI (McNemar-test: *P* = 0.31). Nevertheless, there was a statistically significant difference in diagnosing instability using SR between functional radiographs and SP/MRI (McNemar-test: *P* = 0.025). Calculated values are summarized in Table 5.

**Table 4.** Overview. Lumbar Instability measured by the differences of the sagittal translation (ST ≥ 4 mm) and sagittal rotation (SR ≥ 8°) between FRE (functional radiography extension) and FRF (functional radiography flexion); and between SP (radiography in sagittal plane of the full spine) and MRI. In parentheses, the percentage is presented. Number of lumbar levels are 165 (55 patients and 3 levels). Differences between FRE vs FRF and SP vs MRI were assessed by McNemar-tests.

Lumbar Instability	FRE vs FRF	SP vs MRI	*P*-value
∆ST (≥4 mm)	14 (9)	20 (12)	0.31
∆SR (≥8°)	41 (25)	24 (15)	0.025

**Table 5.** Lumbar instability measured by the absolute differences of the sagittal translation (ST ≥ 4 mm) and sagittal rotation (SR ≥ 8°) between FRE (functional radiography extension) and FRF (functional radiography flexion) (rows); and between SP (radiography in sagittal plane of the full spine) and MRI (columns). Number of lumbar levels are 165 (55 patients and 3 levels). The table shows cross-tabulations over all observations (Vertebral Level = “Total”) and for each vertebral level separately. Differences between FRE vs FRF and SP vs MRI were assessed by McNemar-tests.

Lumbar Instability	Vertrebal Level		No	Yes	*P*-value
∆ST (≥4 mm)	Total	No	136	15	0.307
		Yes	9	5	
	L3/L4	No	51	2	
		Yes	2	0	
	L4/L5	No	39	5	
		Yes	7	4	
	L5/S1	No	46	8	
		Yes	0	1	
∆SR (≥8°)	Total	No	107	17	0.025
		Yes	34	7	
	L3/L4	No	38	1	
		Yes	11	5	
	L4/L5	No	37	5	
		Yes	11	2	
	L5/S1	No	32	11	
		Yes	12	0	

## Discussion

To diagnose vertebral instability, various imaging modalities have been proposed. Due to ease of use, availability, affordability and a short examination time, functional radiographs are the most commonly used imaging method to diagnose lumbar instability and, furthermore, provide a good bone assessment.^24,25^ MRI visualizes ligamentous integrity and a possible fluid gap within the facet joints. CT scans allow three-dimensional visualization of the lumbar spine and have a higher resolution for bony details. Although these modalities are commonly used, no standardized imaging modalities are currently available for diagnosing vertebral instability. Hence, numerous X-ray positions to reveal the maximal displacement of the lumbar body have been introduced.^13,26,27^ Although there hasn’t been a consensus in the literature, flexion-extension radiography is commonly used. However, the number of studies comparing functional radiography to imaging methods like standing X-ray in sagittal plane and MRI, which are carried out as part of the diagnostic process anyway, is rare. Due to the inconsistency of existing data, the necessity of functional radiography in the diagnosis of lumbar instability, compared to standing radiographs and MRI alone, remains unknown.

Kashigar et al. performed a retrospective multicenter study of 191 patients with a grade 1 degenerative spondylolisthesis undergoing surgery, assessing whether additional lumbar motion could be detected through functional radiographs compared to supine MRI and SP alone. In 31 patients, additional motion was seen on flexion-extension radiographs, of which 12 patients had slips >7 mm depicting indication for lumbar body fusion.^28^

Liu et al. did not find any statistically significant difference in diagnosing lumbar instability when comparing functional radiographs to SP and MRI in their prospective study of 68 patients.^14^

Pieper et al. showed an excellent correlation between FRE and SP for translational dislocation in 87 patients with lumbar spondylolisthesis, and concluded omitting extension radiography in routine workup.^29^ Furthermore, prolonged lying during MRI performance might result in the relaxation of paravertebral muscles. Hence, we hypothesized that lumbar flexion takes place, which might be comparable to the flexion in FRF.

In this study, the angular and translational displacement measurements of the upright radiography in the sagittal plane of the full spine are compared to those of the extension radiography, and the ST and SR of the MRI to those of the flexion radiography.

A moderate to excellent agreement between the sagittal translation measured in the SP and the FRE was shown in all analyzed vertebral bodies. This implies that SP can be equally used and thereby substitute the FRE, which is well in line with the literature.^29^ Interestingly, the slip value measured on SP tended to be higher than on FRE, even though this was not statistically significant.

The comparison between the sagittal translation measured in the FRF and MRI showed moderate to good agreement. The FRF gave statistically significantly higher values than the MRI. We figure that although examination methods are well comparable and muscle relaxation in the lying MRI position takes place, axial forces shift the vertebral bodies stronger and display instability to a higher extent. Furthermore, the range of motion of lumbar flexion is physiologically greater than that of lumbar extension, which could also explain the greater range of sagittal translation during flexion. Only five segments were classified as unstable with both measurement methods. A possible explanation for this could be the divergent muscular tension between the respective examination modalities or the patient’s fear of pain to move deep into bending. Beyond that, a longer time interval between the examinations and parameters that were not recorded (such as discomfort at the time of imaging, taking painkillers, etc.) could also be conceivable causes.

However, segmental instability, according to sagittal translation, was diagnosed more often using SP/MRI than with FRF/FRE. Therefore, we conclude that the FRF might give additional information in certain cases but is no longer necessary in routine work-up.

Regarding sagittal rotation, the ICCs revealed moderate agreement between SP and FRE in all three vertebral levels. Only poor agreement between the MRI and FRF was shown. FRF showed significantly lower values than the MRI. We consider muscle relaxation due to the lying position in the MRI as causal.

Another explanation is that the axial load during an FRF may lead to a larger angle between two vertebral bodies and thereby cause greater pressure on nerve roots, resulting in severe pain. Hence, less flexion might be reached in patients with symptomatic lumbar instability. However, this hypothesis would be contrary to the findings in sagittal translation of MRI and FRF.

Regarding sagittal rotation, lumbar instability was diagnosed significantly more often. This discrepancy in the measured angles might be due to the commonly acknowledged measuring inaccuracy of five degrees. However, it has been previously shown that rotational hypermobility (SR) does not directly correlate with the severity of symptoms and, thus, does not justify surgical intervention alone.^17,30^ Radicular symptoms and low back pain with radiographically proven translational instability are considered indications for spinal fusion.^2,4,5,31^ Additionally, it would also be useful in the future to adjust the surgical approach based on the degree of instability, which did not take place in this study due to its retrospective nature.

To the best of our knowledge, no study on lumbar rotational instability suggesting surgical treatment (e.g. spinal fusion) has been published to date (PubMed MeSH: Sagittal Angulation, Lumbar Spinal Fusion, Sagittal Rotation).

This study has several limitations. Certainly, one weakness is the small sample size. Another limitation might be the fact that seven patients with a true spondylolisthesis are included in this study. It is well-known that this kind of disease has a different pathogenesis than degenerative spondylolisthesis. Those patient cohorts generally differ regarding symptoms, age and general health state. The mixture of both conditions presents a potential bias, which was emphasized in the studies by Wood et al.^8^ and Cabraja et al.^26^ Nevertheless, we focussed on measurements for diagnosing sagittal instability in patients with low-grade spondylolisthesis in this study. Therefore, the pathogenesis should not influence the outcome.

Finally, radiographs and MR imaging can be difficult to compare because of varying magnifications. Without a visible calibration, the measurements of structures can be a substantial source of error. Irregular shapes of vertebral bodies can impede a precise measurement within one or multiple imaging methods as well.^32^ However, to avoid inaccuracies due to magnifications, the percentage of the body width is calculated and compared.^33^ In our results, absolute and relative values do not differ significantly and do not change ICC-related reliability outcomes.

Based on our findings, vertebral instability can be equally diagnosed by measuring the difference between the sagittal translation of the SP and MRI in most cases. Hence, the performance of functional imaging in diagnosing vertebral instability, especially the FRE, in the routine work-up, is not needed. Patients can avoid radiation exposure, save time, and receive adequate therapy earlier resulting in an overall shortened period of pain. Furthermore, socioeconomic costs for the health care system can be reduced. However, some borderline cases with severe clinical symptoms might not show a significant slip (ST) between SP and MRI. In those patients, functional radiography in flexion might be an asset for diagnosing instability due to the significantly higher values compared to the MRI.

The assessment of sagittal rotation does not show sufficient agreement between the above-mentioned imaging modalities.

## Conclusion

A vertebral instability of the lumbar spine may be measured by calculating the difference in the sagittal translation in the MRI and the SP. Our data indicate that functional radiographs may no longer be necessary, and waiving such imaging could reduce radiation exposure and save money and time. However, in borderline cases with appropriate symptoms lacking radiological proof for instability, functional radiography in flexion might be an additional diagnostic tool for diagnosing lumbar instability.
